# Supplementary material for: Protein Disulfide Isomerase A3 (PDIA3): A Pharmacological Target in Glioblastoma?
Source: Int J Mol Sci. 2023 Aug 26;24(17):13279. doi: 10.3390/ijms241713279 (PMC10488224; doi:10.3390/ijms241713279)
Supplement: Supplementary file 1 [file ijms-24-13279-s001.zip › ijms-2541217-supplementary.pdf]

Methods

The gene expression profile of GSE147352 (https://doi.org/10.1016/j.molcel.2021.01.015) was downloaded from the Gene Expression Omnibus (GEO; http://www.ncbi.nlm.nih.gov/geo/) database (doi: 10.1007/978-1-4939-3578-9_5). The study comprised 85 adult glioblastomas, 18 lower grade gliomas, and 15 normal brain tissues, characterized by rRNA-depleted total RNAseq. Gene expression data analysis have been conducted using Python equipped with the scipy (10.1038/s41592-019-0686-2) and numpy (https://doi.org/10.1038/s41586-020-2649-2) libraries. PDIA3 expression levels in glioblastoma and normal tissue samples were compared

Results

To investigate the implication of PDIA3 in glioblastoma, we analyzed the gene expression data of glioblastoma in the GSE147352 dataset. Results indicated that the mRNA expression of PDIA3 was significantly increased in the glioblastoma samples in comparison to the normal brain tissue ones (Figure S1). Glioblastoma tissue showed statistically higher PDIA3 expression than normal tissue (both $P<0.0001$), with mean values of 8.14±0.48 and 7.5±0.24. These data indicate that the mRNA expression of PDIA3 is upregulated in glioblastoma than in normal brain tissue.

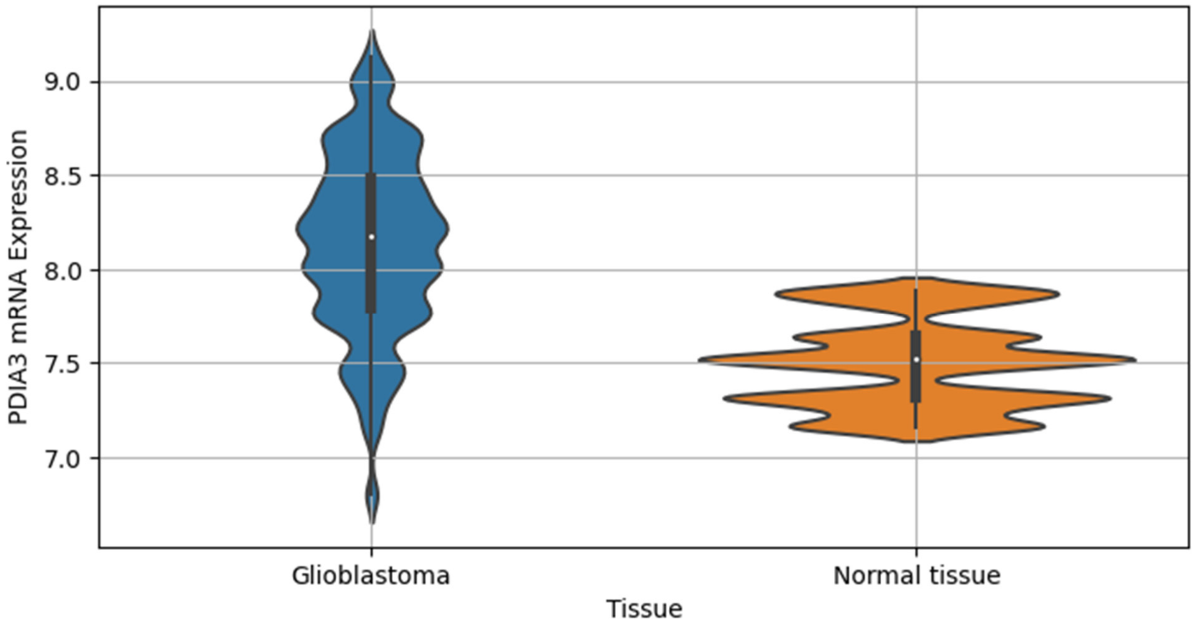

Figure S1. Expression of PDIA3 in glioblastoma and normal brain tissue samples of the GSE147352 samples.
